# Supplementary material for: Elucidating the genetic mechanisms governing cytosine base editing outcomes through CRISPRi screens
Source: Nat Commun. 2025 May 20;16:4685. doi: 10.1038/s41467-025-59948-z (PMC12092667; doi:10.1038/s41467-025-59948-z)
Supplement: Supplementary file 1 — Supplementary Information [file 41467_2025_59948_MOESM1_ESM.pdf]

Supplementary Information for:

Elucidating the genetic mechanisms governing cytosine base editing outcomes through CRISPRi screens

Sifeng Gu, Zsolt Bodai, Rachel A. Anderson, Hei Yu Annika So, Quinn T. Cowan, and Alexis C. Komor

Supplementary Figure 1	Development of C•G to T•A and C•G to G•C fluorescent reporters and deaminase selection.
Supplementary Figure 2	CRISPRi and SaCBE constructs and sgRNA library composition.
Supplementary Figure 3	Example FACS plots and sequencing of the reporter vector from the C•G to T•A and C•G to G•C screens.
Supplementary Figure 4	Screen quality control data.
Supplementary Figure 5	Correlation between replicates of both screens.
Supplementary Figure 6	Genes selected for validation
Supplementary Figure 7	Editing efficiencies and Log_2 Fold Change values following knockdown of gene hits selected from the C•G to T•A screen.
Supplementary Figure 8	Editing efficiencies and Log_2 Fold Change values following knockdown of gene hits selected from the C•G to G•C screen.
Supplementary Figure 9	Distributions of editing outcomes upon selected gene knockdown at reporter vectors.
Supplementary Figure 10	Knockdown effects of selected hits on C•G to G•C and C•G to A•T outcomes.
Supplementary Figure 11	Confirmation of gene knockdown and distributions of relative editing outcomes for CRISPRi K562 cell lines used in endogenous site validation.
Supplementary Figure 12	Validation of hits in K562 cells via transient transfection of rA1-SaBE4(ΔUGI), rA1-SaBE4, or rA1-SaBE1.
Supplementary Figure 13	Validation of hits in HeLa cells via transient transfection of rA1-SaBE4(ΔUGI) or rA1-SaBE4.
Supplementary Figure 14	Validation of hits in HEK293T cells via transient transfection of rA1-SaBE4(ΔUGI), rA1-SaBE4, or evo-BE4 (ΔUGI).
Supplementary Figure 15	Impacts of UNG, MSH2, LIG3, RFWD3, and ERCC4 knockdown on all editing outcomes in HeLa cells.
Supplementary Discussion	Validation of CRISPRi screens<br>Identification of genes that are synthetic lethal to CBE expression and/or activity<br>Further discussion on certain hits below the z-score threshold
Supplementary Figure 16	Identification of genes that are synthetic lethal to CBE expression and/or activity.

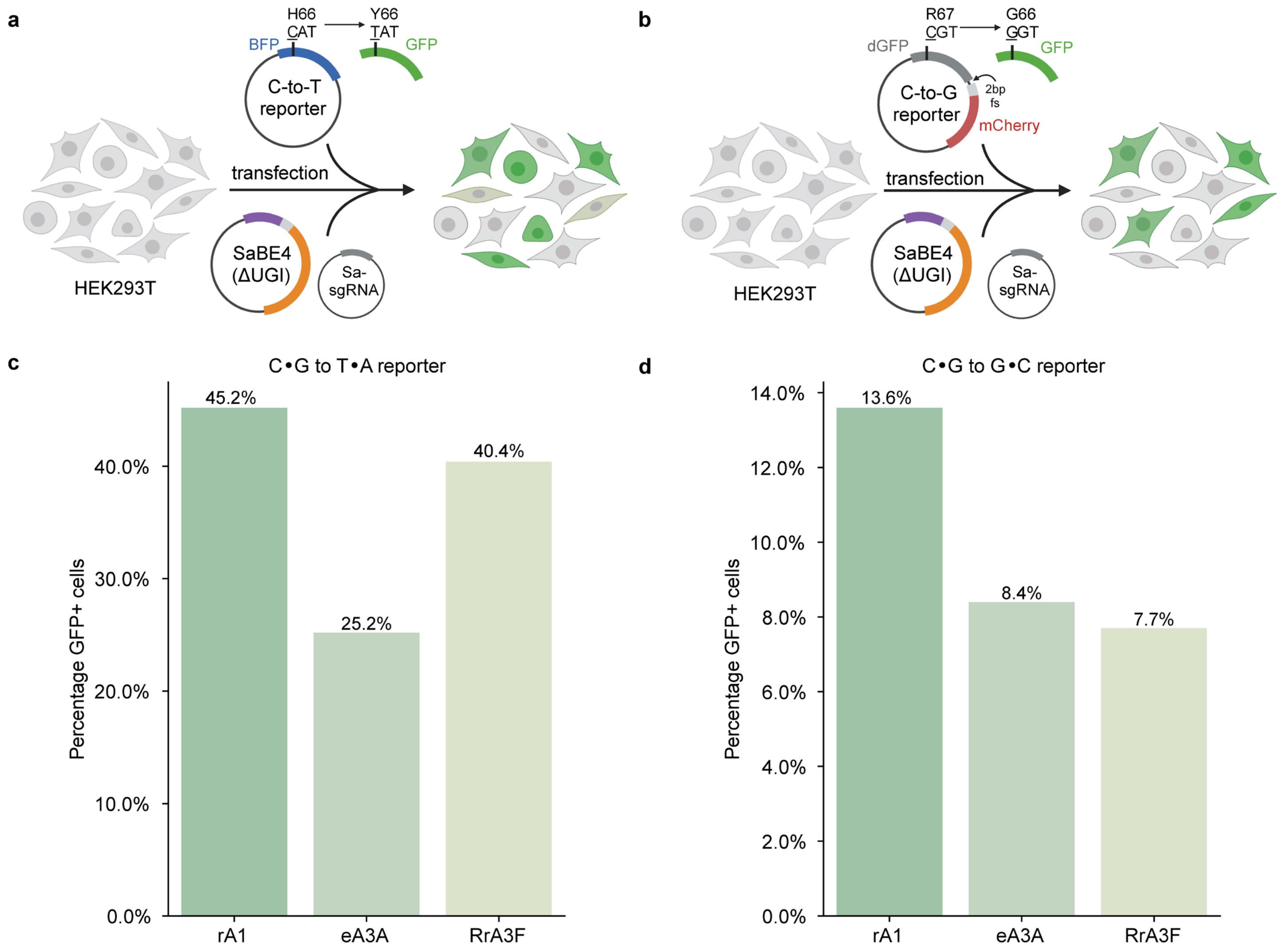

Supplementary Figure 1. Development of C•G to T•A and C•G to G•C fluorescent reporters and deaminase selection. (a-b) HEK293T cells were transfected with either a C•G to T•A reporter (**a**) or a C•G to G•C reporter (**b**), along with a SaCas9-BE4(ΔUGI) construct and a sgRNA targeting the CBE to the mutation of interest. The SaCas9-BE4(ΔUGI) construct consists of the rAPOBEC1, RrA3F, or eA3A deaminase fused to the PAM-relaxed KKH *Staphylococcus aureus* Cas9n (SaCas9n) via an optimized 32 amino acid linker. In the C•G to T•A reporter (**a**), a codon mutation from TAT to CAT generates a Tyr66His mutation in GFP that shifts the protein's fluorescence emission from 509 nm to 445 nm. In the C•G to G•C reporter (**b**), a GGT to CGT codon mutation installs a Gly67Arg mutation in GFP which abolishes all fluorescence. Further, the introduction of indels that cause 2-bp frameshifts restore the reading frame and, thus, the expression of mCherry. Created in BioRender. Gu, S. (2025) https://BioRender.com/849kgfu. (**c-d**) Plotted are the percentages of GFP+ cells at 72 hours post-transfection using flow cytometry. 10,000 events were collected for each sample. Bars show the value of a single replicate. Source data are provided as a Source Data file.

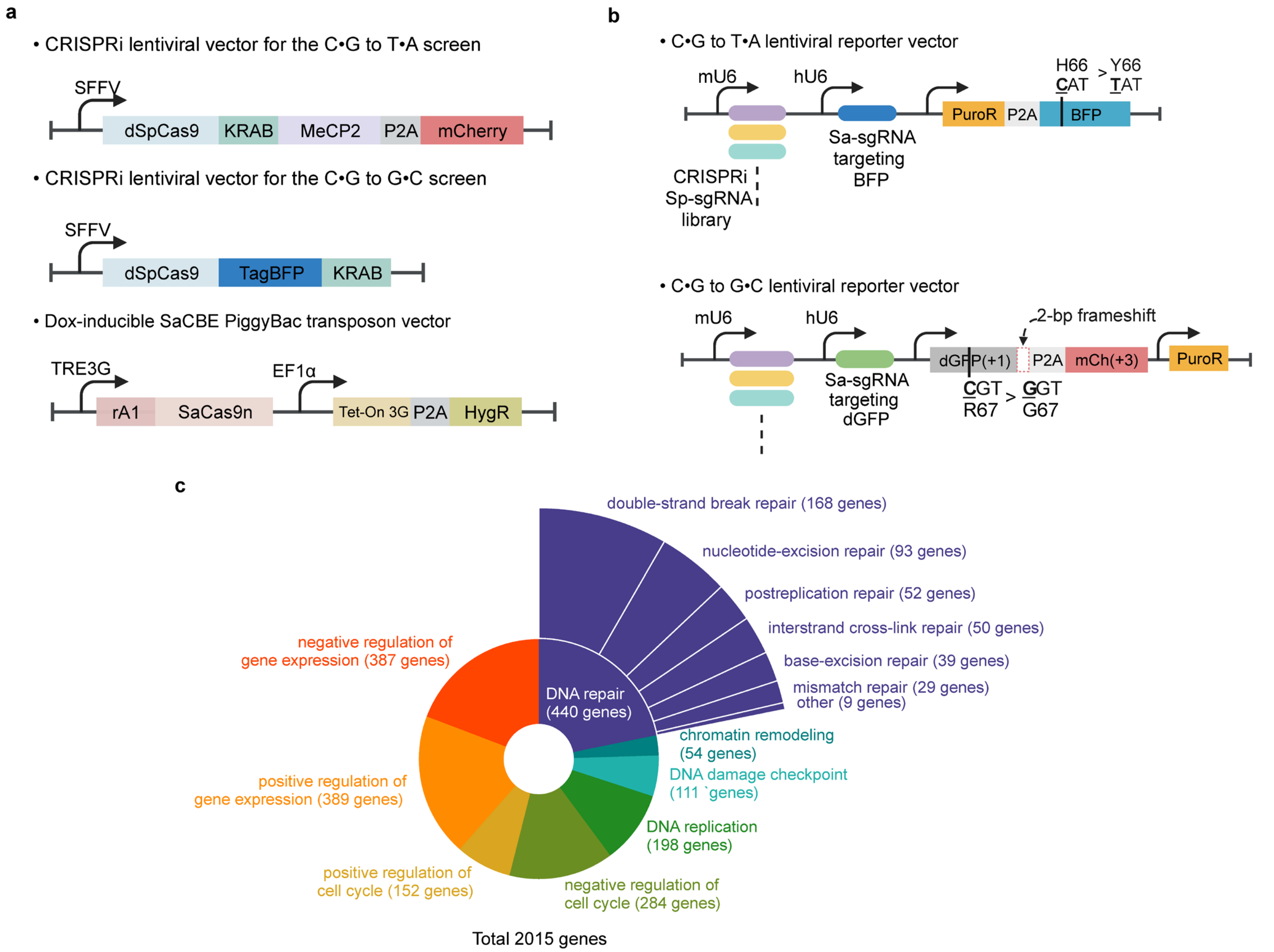

Supplementary Figure 2. CRISPRi and SaCBE constructs and sgRNA library composition. (a) Schematic of vectors used in constructing CRISPRi-expressing K562 cells with dox-inducible rA1-SaBE4(ΔUGI)**.** The CRISPRi construct used for the C•G to T•A screen was comprised of dCas9-KRAB-MeCP2-P2A-mCherry (top), and that for the C•G to G•C screen was comprised of dCas9-BFP-KRAB (middle), allowing for enrichment of successfully transduced cells with fluorescence activated cell sorting (FACS). These fluorescent proteins were chosen so that they would not overlap with their corresponding reporter's fluorescence spectra in future experiments. Doxycycline-inducible SaCBE expression and hygromycin resistance cassettes (bottom) were then incorporated into each CRISPRi-expressing cell line via PiggyBac transposition. A hygromycin selection enabled selection for cells with successful integration. **(b)** Schematic of the C•G to T•A (top) and C•G to G•C (bottom) lentiviral reporter vectors. Each system contains a 12,318-member CRISPRi Sp-sgRNA library (detailed in **c**) that targets the CRISPRi machinery to a different gene of interest for knockdown, a Sa-sgRNA for the rA1-SaBE4(ΔUGI), a puromycin resistance gene, and a fluorescent protein gene that undergoes a phenotypic change (blue to green fluorescence change for the C•G to T•A reporter, no color to green fluorescence for the C•G to G•C reporter) upon cytosine base editing. **(c)** Composition and functional annotations of the CRISPRi sgRNA library. Shown are the gene ontology (GO) terms for the genes targeted by the CRISPRi sgRNA library. The library targets 2,015 genes (and is comprised of 12,318 sgRNAs, including 5 sgRNAs per gene and 2,243 non-targeting sgRNAs) related to DNA metabolic processes and the cellular response to DNA damage. Notably, this library includes 440 DNA repair genes which cover all DNA repair pathways, and 57 essential genes to serve as controls.[1–3] Source data are provided as a Source Data file. Created in BioRender. Gu, S. (2025) https://BioRender.com/gtw43wi.

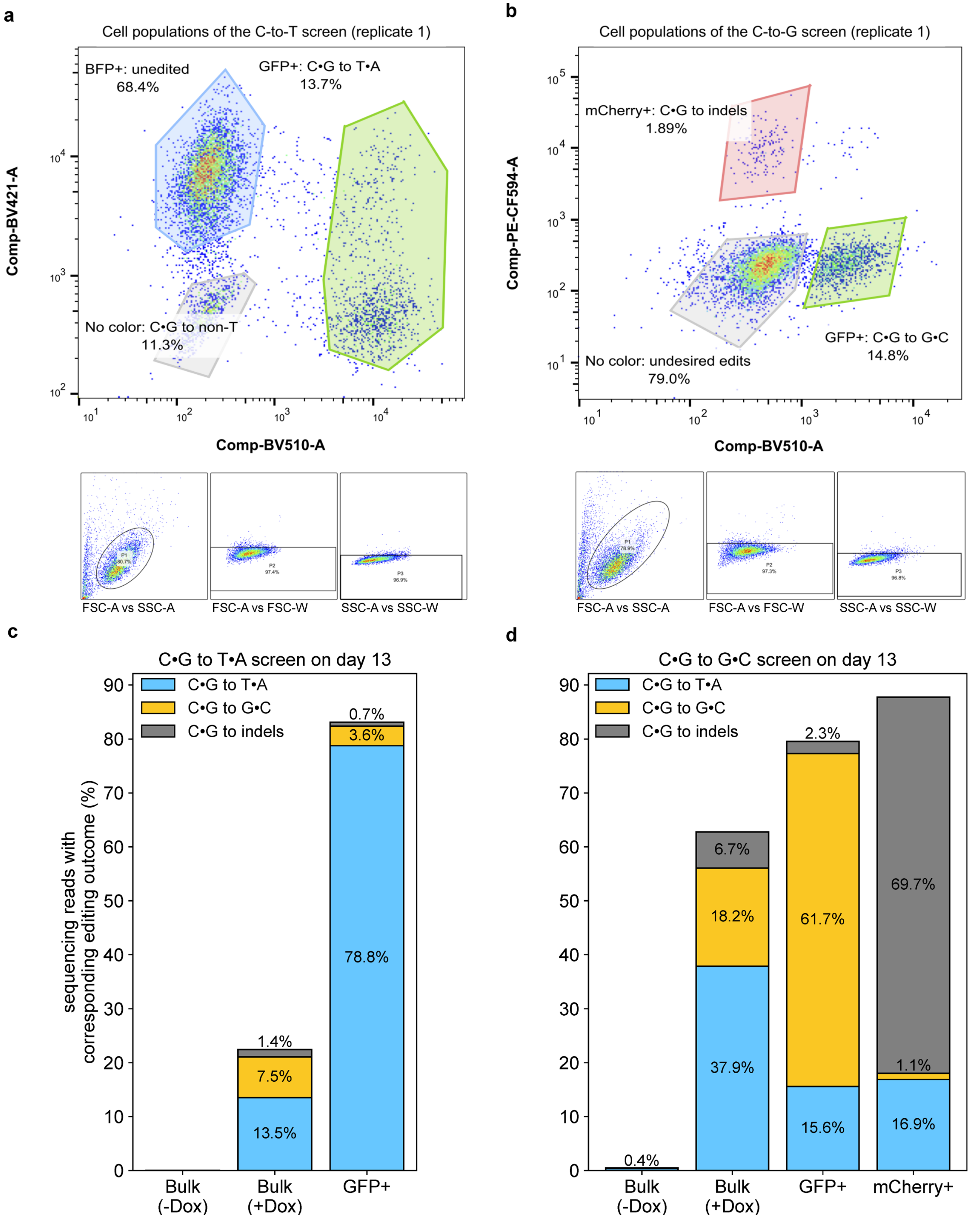

Supplementary Figure 3. Example FACS plots and sequencing of the reporter vector from the C•G to T•A and C•G to G•C screens. Screens were run as described in Figure 1c. **(a-b)** FACS plots of replicate 1 (out of n=2 independent experiments) of the C•G to T•A (**a**) and C•G to G•C (**b**) screens are shown. Live cell gates (P1,

P2 and P3) are shown in the three subplots under the main plot. Percentages in each gate are derived from 10,000 events. **(c-d)** Next Generation Sequencing (NGS) data of replicate 1 of the C•G to T•A (**c**) and C•G to G•C (**d**) screens on day 13 are shown. Percentage of total DNA sequencing reads with C•G to T•A, C•G to G•C, and C•G to indel outcomes in each sample are plotted. Bulk (-Dox) samples are those in which no rA1-SaBE4(ΔUGI) expression was induced. Bulk (+Dox) samples are those in which rA1-SaBE4(ΔUGI) was induced for 13 days, but cells were not sorted. GFP+ samples are those in which rA1-SaBE4(ΔUGI) was induced for 13 days and cells from the GFP+ populations (green gates in **a** and **b**) were collected via FACS. The mCherry+ sample is that in which rA1-SaBE4(ΔUGI) was induced for 13 days and cells from the mCherry+ population (red gate in **b**) were collected via FACS. We note that the C•G to indel rate in the unsorted control population is higher than what was quantified by flow cytometry because not all indels cause a 2-bp frameshift. Source data are provided as a Source Data file.

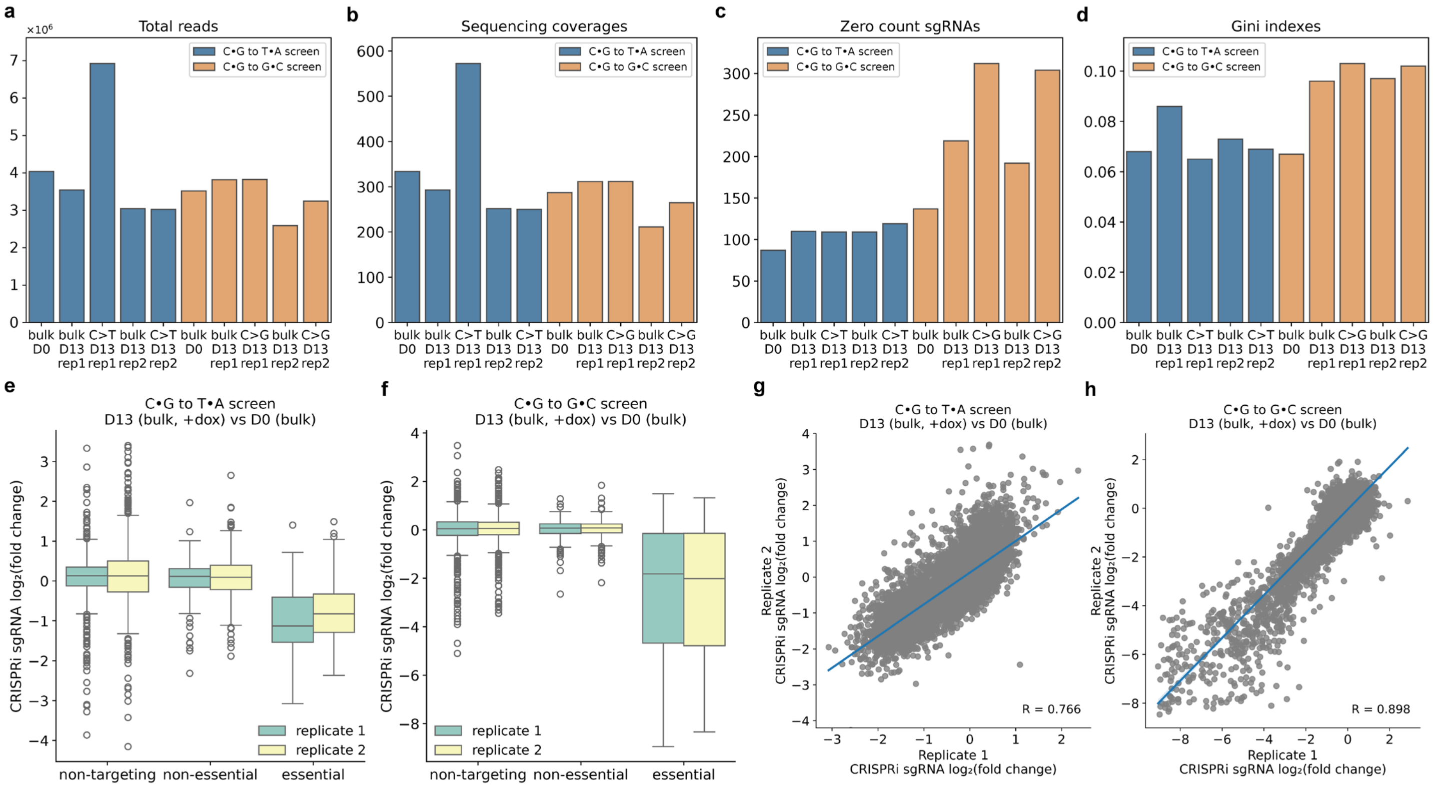

Supplementary Figure 4. Screen quality control data. Screens were run as described in Figure 1c. (**a-d**) Screen quality controls. Shown are number of total NGS reads (**a**), sequencing coverages (**b**), number of zero-count CRISPRi sgRNAs (**c**), and Gini indexes (**d**) for all populations obtained for the screens. **(e-f)** sgRNAs targeting essential genes are depleted over time in both screens. Shown in the boxplots are the distributions of $\log_2$ fold changes of CRISPRi sgRNAs in each replicate of the C•G to T•A (**e**) and C•G to G•C screen (**f**), when comparing the bulk unsorted samples in the experimental arm (+dox) on day 13 (D13) to their corresponding initial day 0 (D0) samples. CRISPRi sgRNAs are categorized by non-targeting, non-essential and essential. Center line, median; box, interquartile range; whisker, up to 1.5x interquartile range; Outliers are shown individually. **(g-h)** Strong correlations were observed between the two replicates in each screen. Shown in the scatterplots are the correlations between the $\log_2$ fold changes of CRISPRi sgRNAs of the two replicates. The bulk unsorted samples in the experimental arm (+dox) on day 13 (D13) are compared to their corresponding initial day 0 (D0) samples. The blue lines represent the fitted linear regression, and Pearson's r values are annotated. Source data are provided as a Source Data file.

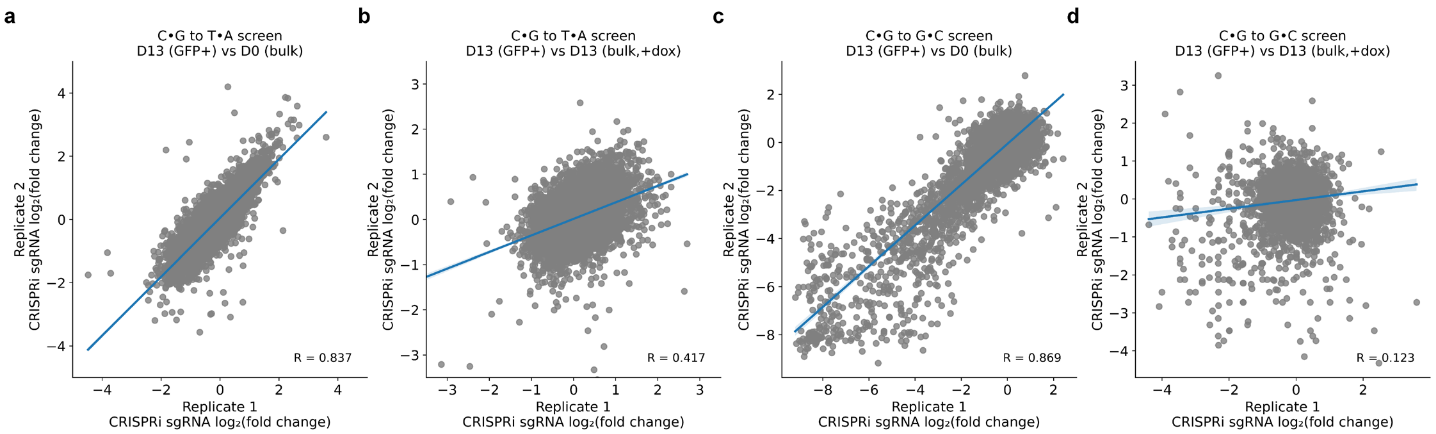

Supplementary Figure 5. Correlation between replicates of both screens. Screens were run as described in Figure 1c. **(a and c)** Shown in the scatterplots are the correlations between the two replicates of the C•G to T•A (**a**) and C•G to G•C (**c**) screen, comparing the GFP+ population on D13 to the bulk population on D0. Log2 fold changes of CRISPRi sgRNAs in replicate 1 is plotted against those in replicate 2. Blue line represents the fitted linear regression, and the Pearson's r values are annotated on the bottom right corner. **(b and d)** Correlations between the two replicates of the C•G to T•A (**b**) and C•G to G•C (**d**) screen, comparing the GFP+ population on D13 to the bulk population on D13. Linear regression line and Pearson's r values are also shown. Low correlation between replicates in (**b**) and (**d**) we believe is due to only a small number of DNA processing genes being able to single-handedly impact base editing outcomes, potentially due to DNA repair pathway redundancy. Source data are provided as a Source Data file.

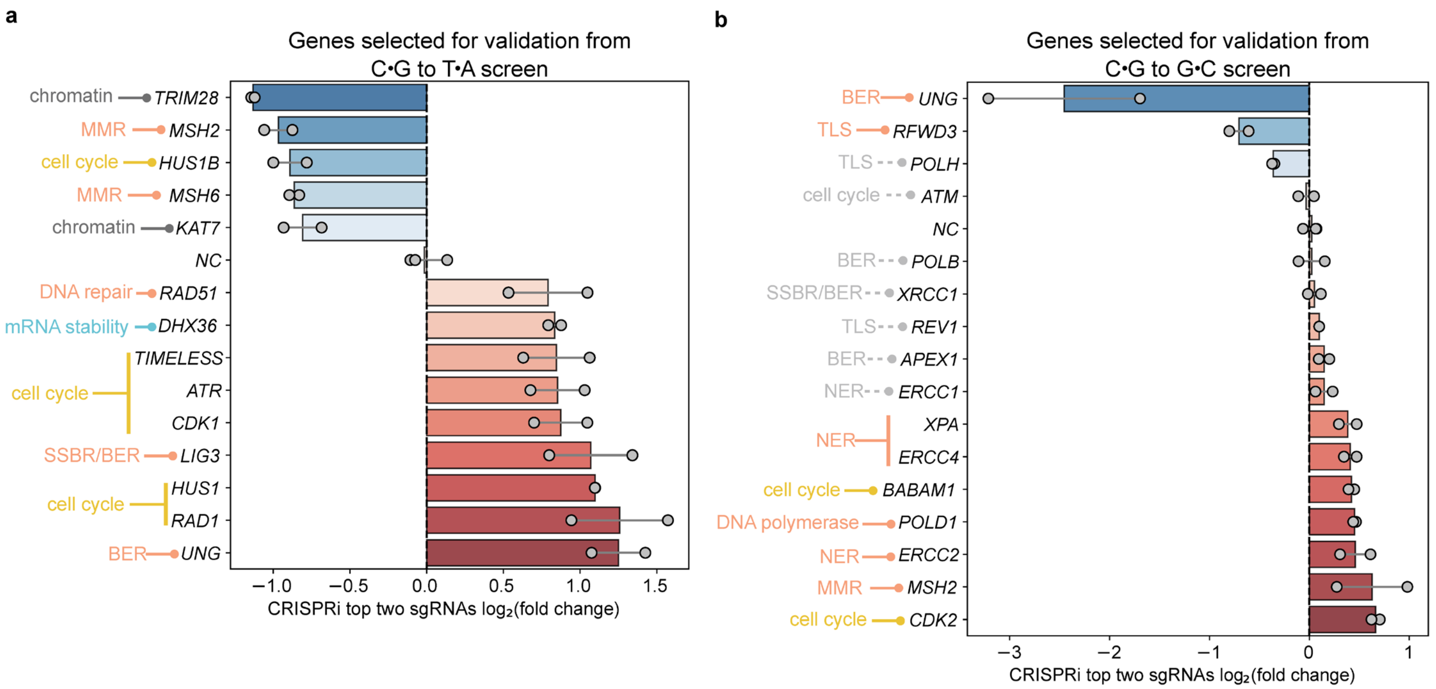

Supplementary Figure 6. Genes selected for validation. (a-b) The log2 fold changes of the top two CRISPRi gRNAs for each gene selected for further validation are shown. The bars represent the mean of the log2 fold change, with lines connecting each sgRNA which is marked individually. The pathways to which each gene belongs are annotated on the left. Hits identified from the screens are linked to their pathways in solid lines with colors consistent with the volcano plots. Genes that are hypothesized to resolve cytosine base editing intermediate, but did not show up as hits from the screens, are linked to their pathways in grey dash lines. MMR, DNA mismatch repair. BER, base excision repair. SSBR, single-stranded break repair. TLS, translesion synthesis. NER, nucleotide excision repair. Source data are provided as a Source Data file.

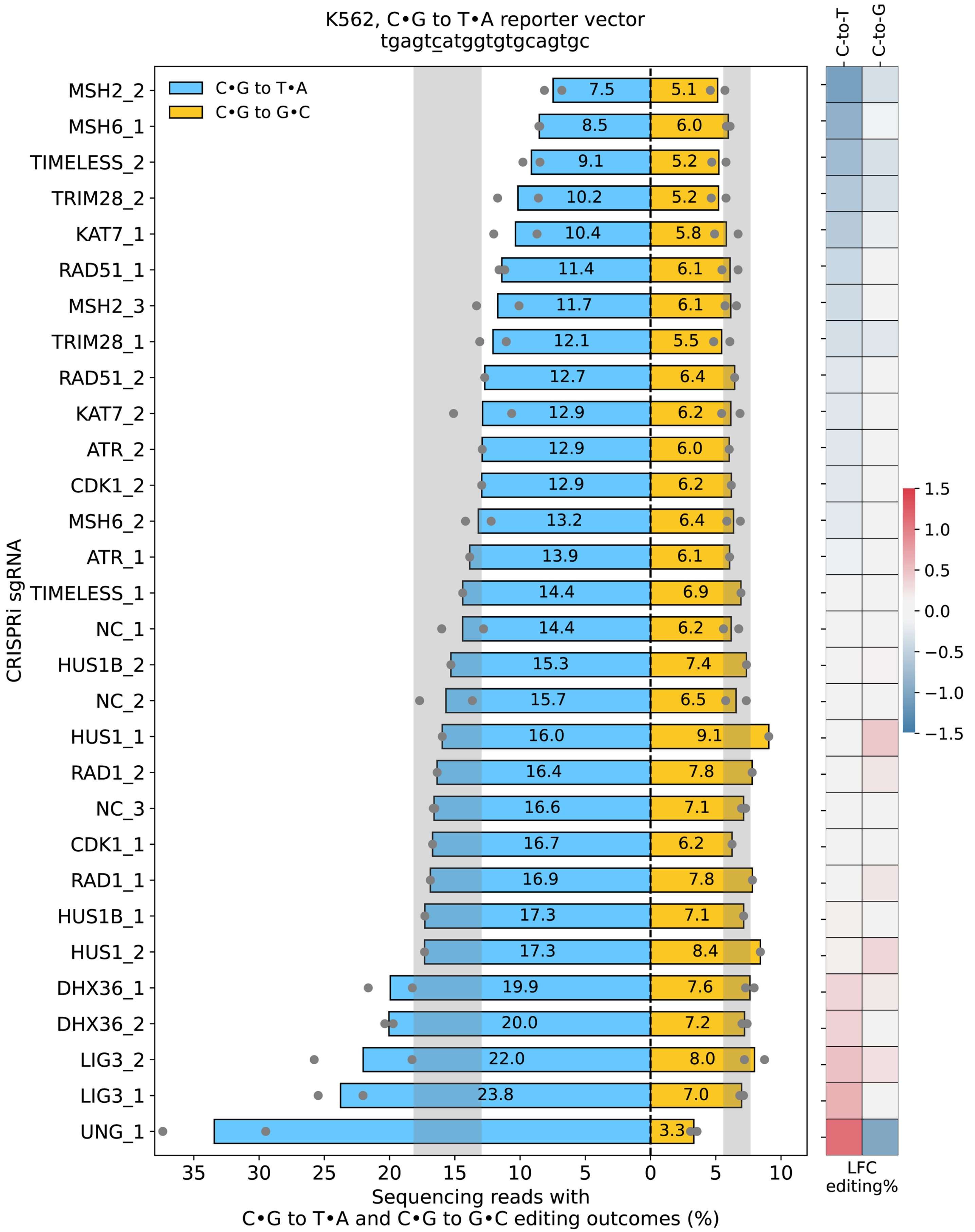

Supplementary Figure 7. Editing efficiencies and Log_2 Fold Change values following knockdown of gene hits selected from the C•G to T•A screen. Individual gene hits were evaluated as described in Figure 2a.

Shown are absolute C•G to T•A and C•G to G•C editing efficiencies at the single-copy, genomically-integrated BFP-based C•G to T•A reporter upon knockdown of selected genes with the two best-performing CRISPRi sgRNAs. For the NC samples, the bars represent the mean obtained from n=3 biological replicates. For certain samples, the bars represent the mean of n=2 biological replicates. For all other samples, the bars represent the value of single experiments. The shaded regions represent the ranges that are within three STDs from the mean C•G to T•A or C•G to G•C editing efficiencies of the NC samples (i.e. values within these ranges are considered not statistically significant). A sgRNA is considered to have a significant impact on base editing when any of its corresponding editing efficiencies fall outside of these ranges. Heatmaps represent $\log_2$ (fold change) (LFC) in C•G to T•A and C•G to G•C editing efficiencies relative to the mean of 3 non-targeting sgRNAs. Source data are provided as a Source Data file.

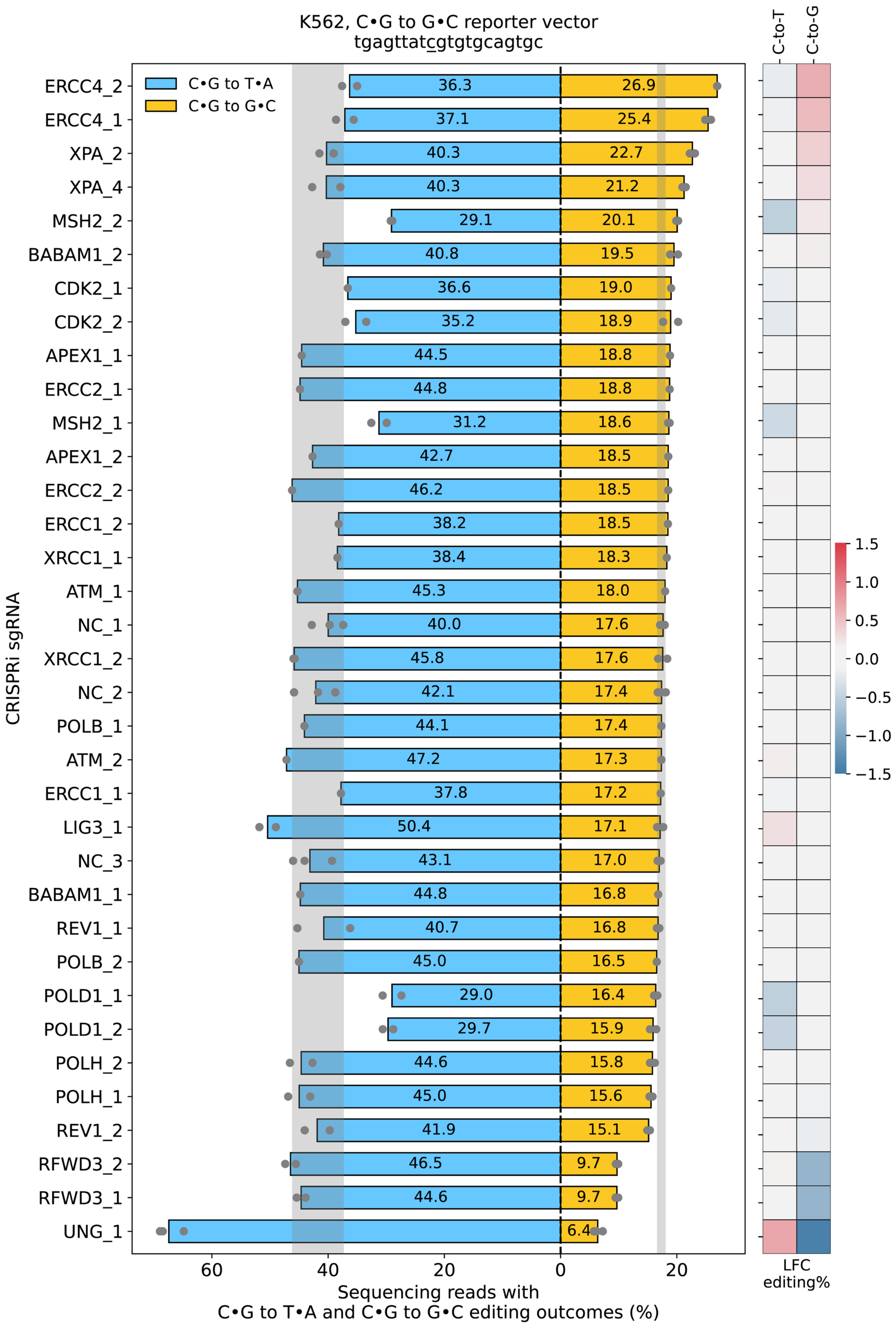

K562, C•G to G•C reporter vector
tgagttatcgtgtgcagtgc
C•G to T•A
C•G to G•C
CRISPRi sgRNA
ERCC4_2 36.3 26.9
ERCC4_1 37.1 25.4
XPA_2 40.3 22.7
XPA_4 40.3 21.2
MSH2_2 29.1 20.1
BABAM1_2 40.8 19.5
CDK2_1 36.6 19.0
CDK2_2 35.2 18.9
APEX1_1 44.5 18.8
ERCC2_1 44.8 18.8
MSH2_1 31.2 18.6
APEX1_2 42.7 18.5
ERCC2_2 46.2 18.5
ERCC1_2 38.2 18.5
XRCC1_1 38.4 18.3
ATM_1 45.3 18.0
NC_1 40.0 17.6
XRCC1_2 45.8 17.6
NC_2 42.1 17.4
POLB_1 44.1 17.4
ATM_2 47.2 17.3
ERCC1_1 37.8 17.2
LIG3_1 50.4 17.1
NC_3 43.1 17.0
BABAM1_1 44.8 16.8
REV1_1 40.7 16.8
POLB_2 45.0 16.5
POLD1_1 29.0 16.4
POLD1_2 29.7 15.9
POLH_2 44.6 15.8
POLH_1 45.0 15.6
REV1_2 41.9 15.1
RFWD3_2 46.5 9.7
RFWD3_1 44.6 9.7
UNG_1 6.4
60 40 20 0 20
Sequencing reads with
C•G to T•A and C•G to G•C editing outcomes (%)
C-to-T
C-to-G
LFC
editing%
1.5
1.0
0.5
0.0
−0.5
−1.0
−1.5

Supplementary Figure 8. Editing efficiencies and Log_2 Fold Change values following knockdown of gene hits selected from the C•G to G•C screen. Individual gene hits were evaluated as described in Figure 2a. Shown are absolute C•G to T•A and C•G to G•C editing efficiencies at the single-copy, genomically-integrated dGFP-based C•G to G•C reporter upon knockdown of selected genes with the two best-performing CRISPRi sgRNAs. For the NC samples, the bars represent the mean obtained from n=3 biological replicates. For certain samples, the bars represent the mean of n=2 biological replicates. For all other samples, the bars represent the value of single experiments. The shaded regions represent the ranges that are within three STDs from the mean C•G to T•A or C•G to G•C editing efficiencies of the NC samples (i.e. values within these ranges are considered not statistically significant). A sgRNA is considered to have a significant impact on base editing when any of its corresponding editing efficiencies fall outside of these ranges. Heatmaps represent log_2 (fold change) (LFC) in C•G to T•A and C•G to G•C editing efficiencies relative to the mean of 3 non-targeting sgRNAs. Source data are provided as a Source Data file.

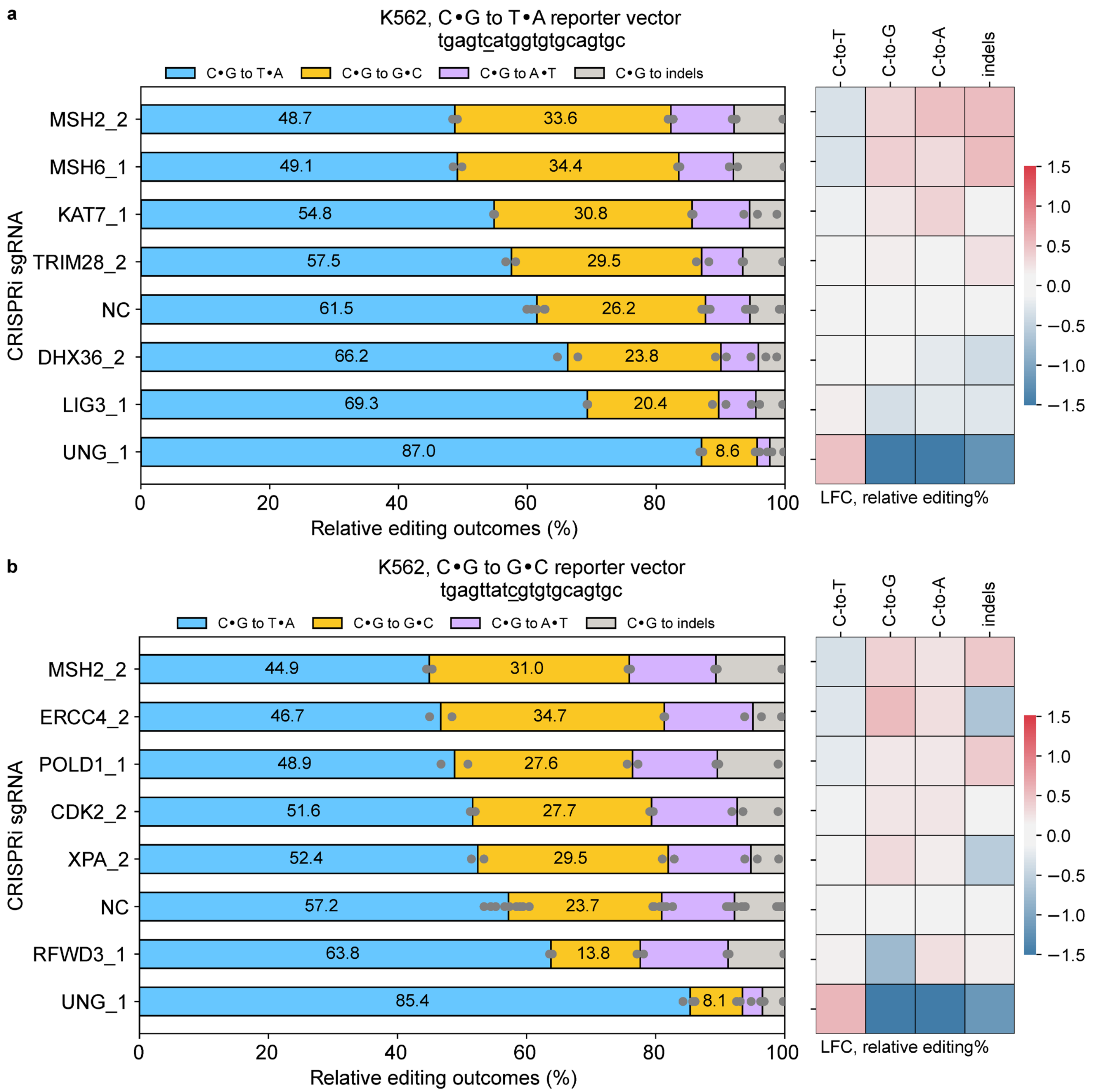

Supplementary Figure 9. Distributions of relative editing outcomes upon selected gene knockdown at reporter vectors. Cells were treated as described in Figure 2a. Shown are the relative percentage of all editing outcomes at the single-copy, genomically-integrated C•G to T•A (**a**) or C•G to G•C (**b**) reporter vectors upon knockdown of selected genes with the best-performing CRISPRi sgRNAs. Relative editing percentages are calculated by normalizing the editing percentages of the outcomes of interest to the total editing percentage, which is the sum of C•G to T•A, C•G to G•C, C•G to A•T and C•G to indel editing efficiencies. Negative control (NC) samples are also shown, in which cells were treated identically using a non-targeting sgRNA. For the NC samples, the bars represent the mean obtained from 3 non-targeting sgRNAs, with n=3 biological replicates each. For all other samples, the bars represent the mean of n=2 biological replicates. Heatmaps represent $\log_2$ (fold change) (LFC) in relative editing percentages of all outcomes relative to the corresponding means of non-targeting sgRNAs. Source data are provided as a Source Data file.

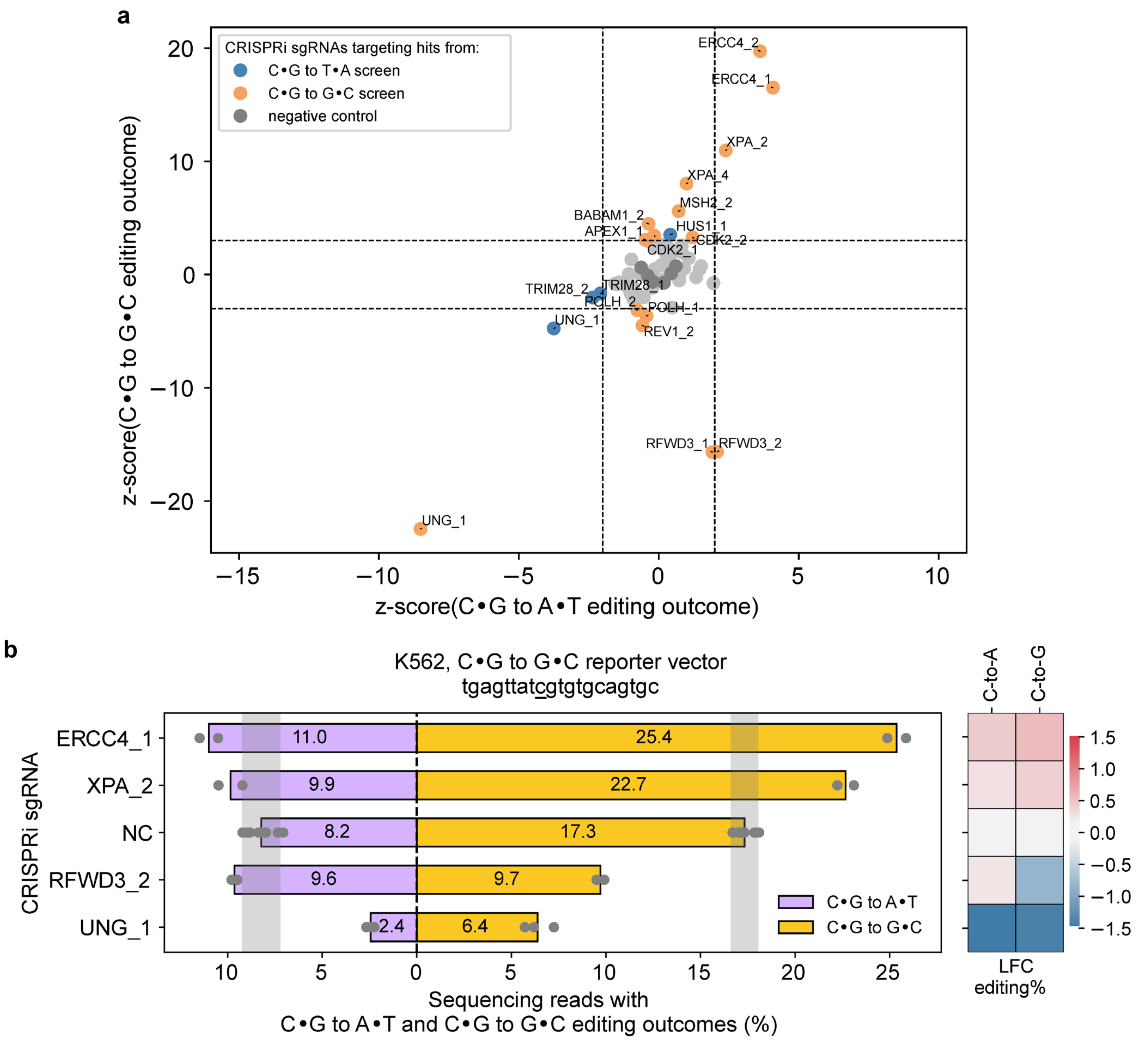

Supplementary Figure 10. Knockdown effects of selected hits on C•G to G•C and C•G to A•T outcomes. Individual gene hits were evaluated as described in Figure 2a. **(a)** Z-scores of changes in C•G to A•T editing efficiencies for each CRISPRi sgRNA (x-axis) were plotted against the z-scores of changes in C•G to G•C editing efficiencies (y-axis). For both screens, sgRNAs that have z-scores higher than the thresholds in either C•G to A•T or C•G to G•C editing outcome (dotted lines: |z-scores(C•G to A•T)| > 2, |z-scores(C•G to G•C)| > 3) are highlighted in color corresponding to the respective screen from which they were considered a hit. **(b)** Absolute C•G to A•T and C•G to G•C editing efficiencies at the single-copy, genomically-integrated *dGFP*-based reporters upon knockdown of selected genes with the best-performing CRISPRi sgRNAs are shown. Negative control (NC) samples are also shown, in which cells were treated identically using a non-targeting sgRNA. For the NC samples, the bars represent the mean obtained from 3 non-targeting sgRNAs, with n=3 biological replicates each. For all other samples, the bars represent the mean of n=2 biological replicates. The shaded regions represent the ranges that are within three standard deviations from the mean C•G to A•T or C•G to G•C editing efficiencies of the NC samples. Heatmaps represent $\log_2$ (fold change) (LFC) in C•G to A•T and C•G to G•C editing efficiencies relative to the corresponding means of non-targeting sgRNAs. Source data are provided as a Source Data file.

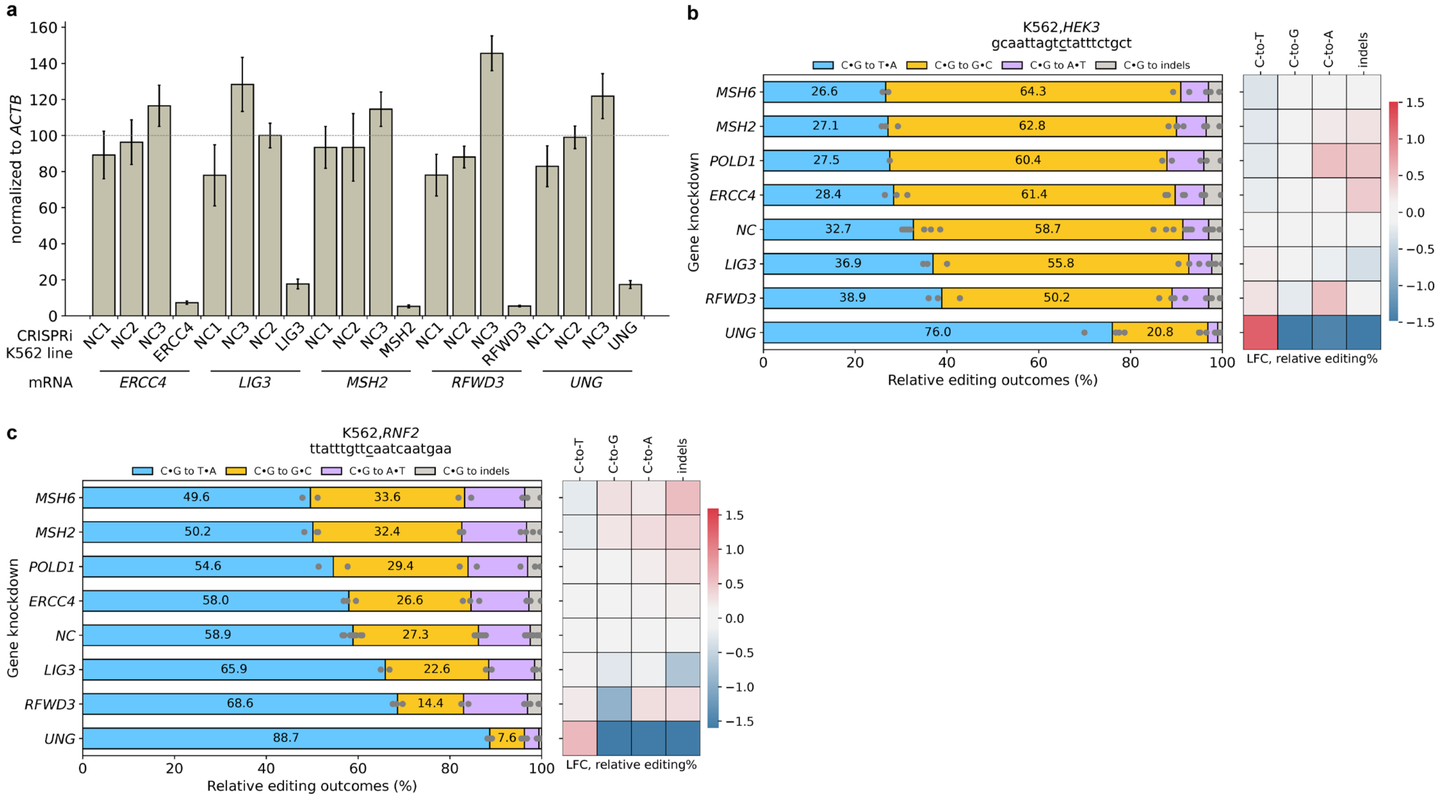

Supplementary Figure 11. Confirmation of gene knockdown by RT-qPCR for CRISPRi K562 cell lines used in endogenous site validation and distributions of relative editing outcomes upon selected gene knockdown. (a) The dCas9-BFP-KRAB and dox-inducible rA1-SaBE4(ΔUGI) K562 cell lines were transduced with the C•G to T•A lentiviral reporter vector (Supplementary Figure 2b) in which the *BFP*-targeting Sa-sgRNA spacer was replaced with either a *HEK3*- or *RNF2*-targeting spacer and the CRISPRi Sp-sgRNA was targeted to the gene indicated. After a 3-day puromycin selection, doxycycline was added and rA1-SaBE4(ΔUGI) expression was induced for 7 days. RNA was then collected, and the mRNA transcripts indicated were measured by RT-qPCR and normalized to *ACTB* mRNA levels. Bars and error bars represent the mean and standard deviation of n=3 replicates. “CRISPRi K562 line” indicates which gene was targeted for knockdown with CRISPRi, where NC1-3 are non-targeting sgRNAs. **(b-c)** Cells were treated as described in Figure 3a-b. Shown are relative percentage of all editing outcomes at the *HEK3* (**b**) or *RNF2* (**c**) sites upon knockdown of selected genes. Relative editing percentages are calculated by normalizing the editing percentages of the outcome of interest to the total editing percentage, which is the sum of C•G to T•A, C•G to G•C, C•G to A•T, and C•G to indels editing efficiencies. Negative control (NC) samples are also shown, in which cells were treated identically using a non-targeting sgRNA. For the NC samples, the bars and the error bars obtained from 3 non-targeting sgRNAs, with n=4 biological replicates each. For all other samples, the bars represent the mean of n=2 (*MSH6* and *POLD1*), 3 (*LIG3*), or 4 (*UNG*, *MSH2*, *RFWD3* and *ERCC4*) biological replicates. Heatmaps represent $\log_2$ (fold change) (LFC) in relative editing percentages of all outcomes relative to the corresponding means of non-targeting sgRNAs. Source data are provided as a Source Data file.

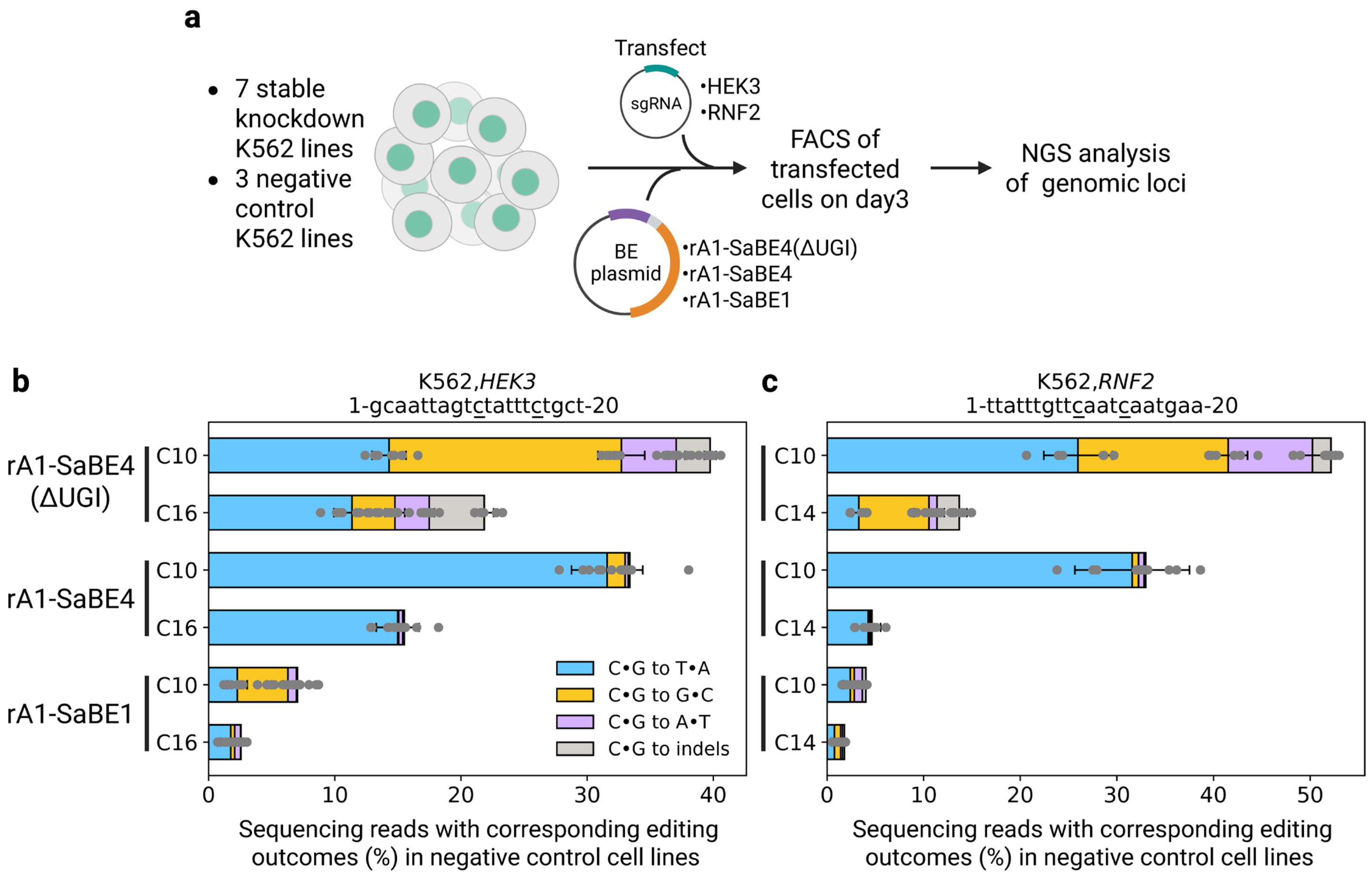

Supplementary Figure 12. Validation of hits in K562 cells via transient transfection of rA1-SaBE4(ΔUGI), rA1-SaBE4, or rA1-SaBE1. (a) CRISPRi K562 stable knockdown cell lines from Figure 2a were transfected with plasmids encoding additional sgRNA and either rA1-SaBE4(ΔUGI), rA1-SaBE4, or rA1-SaBE1. Three days after transfection, transfected cells were collected via FACS and the *HEK3* or *RNF2* locus (site of editing) were sequenced with NGS to quantify editing efficiencies. Created in BioRender. Gu, S. (2025) https://BioRender.com/466q6kr. **(b-c)** Shown are absolute editing efficiencies of all outcomes by rA1-SaBE4(ΔUGI), rA1-SaBE4, or rA1-SaBE1 at the *HEK3* (**b**) or *RNF2* (**c**) sites, broken down by target C within the protospacer, in negative control (NC) samples (non-targeting CRISPRi sgRNAs). The bars and the error bars represent the mean and propagation of uncertainty of standard deviation (STD) obtained from 3 non-targeting sgRNAs, with n=3 and n = 2 biological replicates for *HEK3* and *RNF2*, respectively. Source data are provided as a Source Data file.

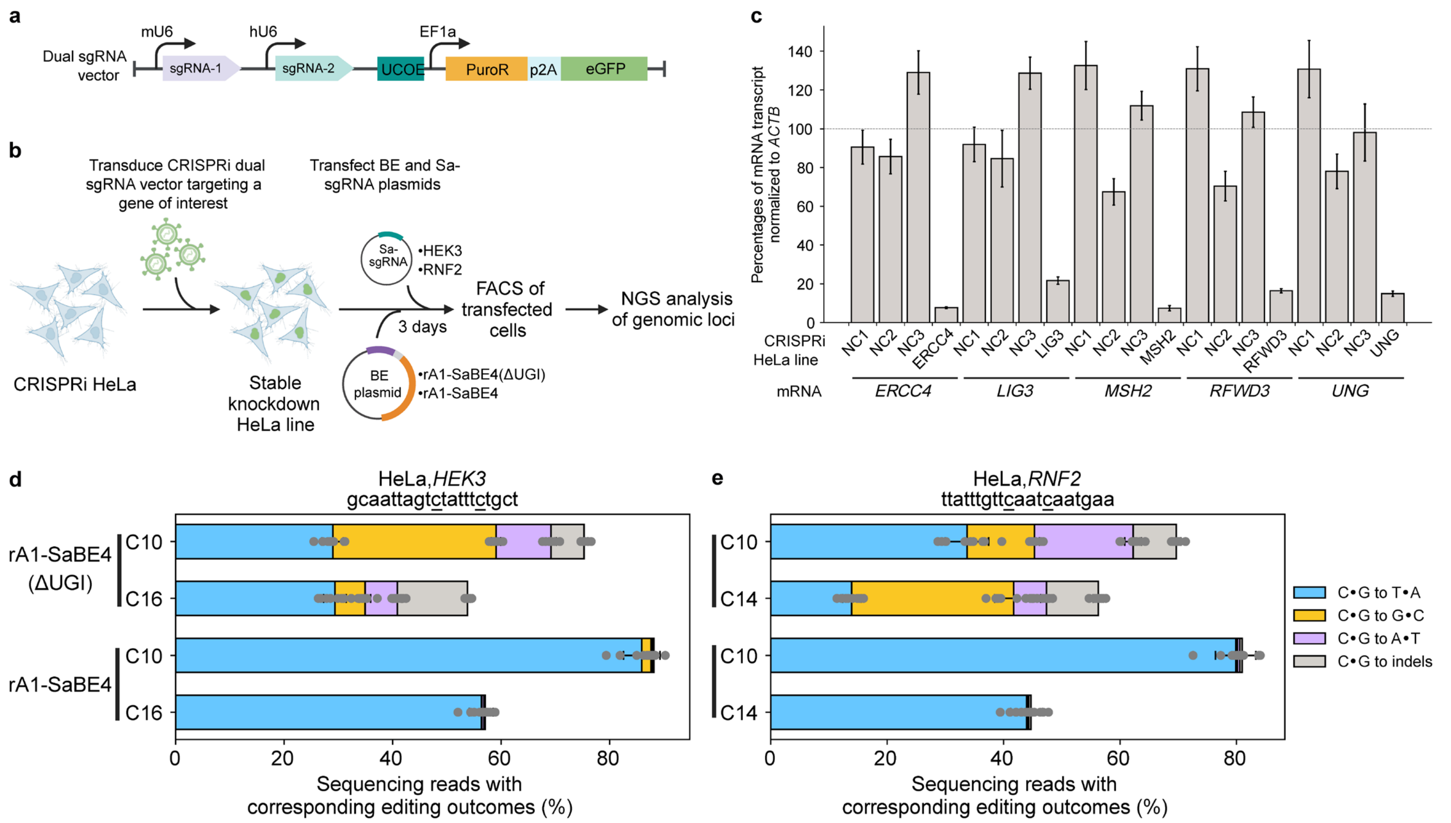

Supplementary Figure 13. Validation of hits in HeLa cells via transient transfection of rA1-SaBE4(ΔUGI) or rA1-SaBE4. (a) Schematic of the dual sgRNA vectors used in the HeLa and HEK293T experiments. (**b**) Schematic of the workflow of validation experiments in HeLa cells. CRISPRi-expressing HeLa cells were transduced with the dual sgRNA vectors targeting selected genes of interest to generate stable knockdown HeLa lines. These cell lines were then co-transfected with either rA1-SaBE4(ΔUGI) or rA1-SaBE4 and Sa-gRNA targeting one of two endogenous sites. Three days post-transfection, transfected cells were collected by FACS and editing efficiencies at the target sites were quantified by NGS. Created in BioRender. Gu, S. (2025) https://BioRender.com/la4yjzm. (**c**) RT-qPCR measurements of target gene knockdown in HeLa cell lines. Bars and error bars represent the mean and standard deviation of n=3 replicates. (**d-e**) Shown are absolute efficiencies of all outcomes by rA1-SaBE4(ΔUGI) or rA1-SaBE4 at the *HEK3* (**d**) and *RNF2* (**e**) sites, broken down by target C within the protospacer, in negative control (NC) samples (non-targeting CRISPRi sgRNAs). The bars and the error bars represent the mean and propagation of uncertainty of standard deviation (STD) obtained from 3 non-targeting sgRNAs, with n=3 biological replicates each. Source data are provided as a Source Data file.

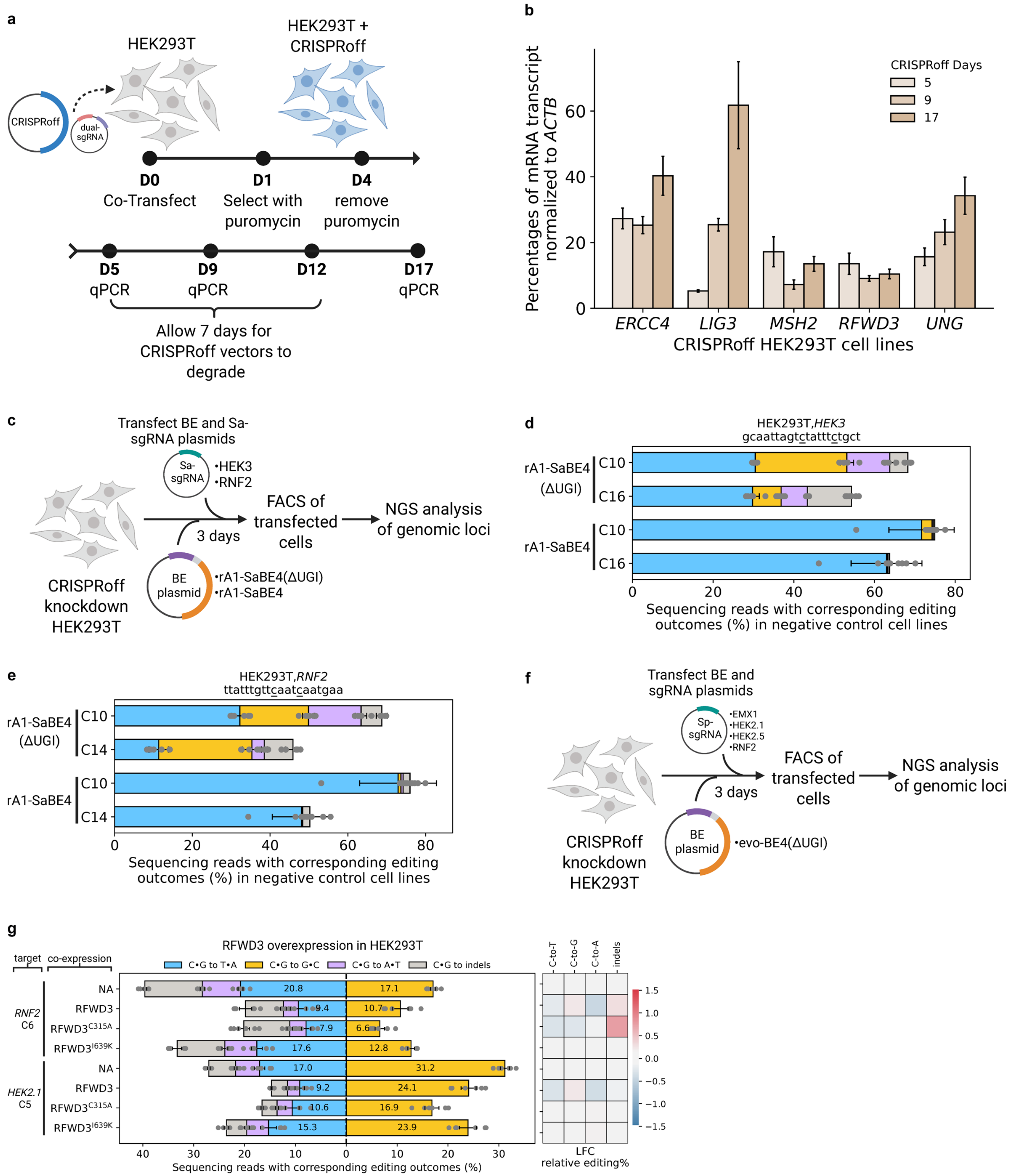

Supplementary Figure 14. Validation of hits in HEK293T cells via transient transfection of rA1-SaBE4(ΔUGI), rA1-SaBE4, or evo-BE4(ΔUGI). (**a**) Generation of HEK293T knockdown cell lines. The CRISPRoff and dual sgRNA vectors targeting selected genes of interest were transiently transfected into wild-type HEK293T cells to generate stable knockdown HEK293T lines. Transfected cells were selected with a 3-day puromycin treatment and were passaged for an additional 7 days to allow for degradation of the CRISPRoff and dual sgRNA vectors. RT-qPCR samples were harvested on D5, D9 and D17. (**b**) RT-qPCR measurements of

target gene knockdown in HEK293T cell lines. Values and error bars represent the mean and standard deviation of n=3 replicates. (**c**) Schematic of the workflow of validation experiments in HEK293T cells. CRISPRoff knockdown HEK293T cells were co-transfected with either rA1-SaBE4(ΔUGI) or rA1-SaBE4 and Sa-gRNA targeting one of two endogenous sites. Three days post-transfection, transfected cells were collected by FACS and editing efficiencies at the target sites were quantified by NGS. (**d-e**) Shown are absolute efficiencies of all outcomes by rA1-SaBE4(ΔUGI) or rA1-SaBE4 at the *HEK3* (**d**) and *RNF2* (**e**) sites, broken down by target C within the protospacer, in negative control (NC) samples (non-targeting CRISPRoff sgRNAs). The bars and the error bars represent the mean and propagation of uncertainty of standard deviation (STD) obtained from 2 non-targeting sgRNAs, with n=3 biological replicates each. (**f**) CRISPRoff knockdown HEK293T cells were co-transfected with evo-BE4(ΔUGI) and Sp-gRNA targeting one of four endogenous sites. Three days post-transfection, transfected cells were collected by FACS and editing efficiencies at the target sites were quantified by NGS. (**g**) Shown are editing efficiencies of all outcomes at two endogenous targets upon overexpression of these mutants in wild-type HEK293T cells. The bars and the error bars represent the mean and propagation of uncertainty of standard deviation (STD) obtained from n=4 biological replicates. Negative control (NA) samples are also shown, in which cells were treated identically without compensation or overexpression. Heatmap shows $\log_2$ (fold change) (LFC) in relative percentages of editing outcomes relative to the corresponding means of negative controls. Source data are provided as a Source Data file. Created in BioRender. Gu, S. (2025) https://BioRender.com/frx2mnr.

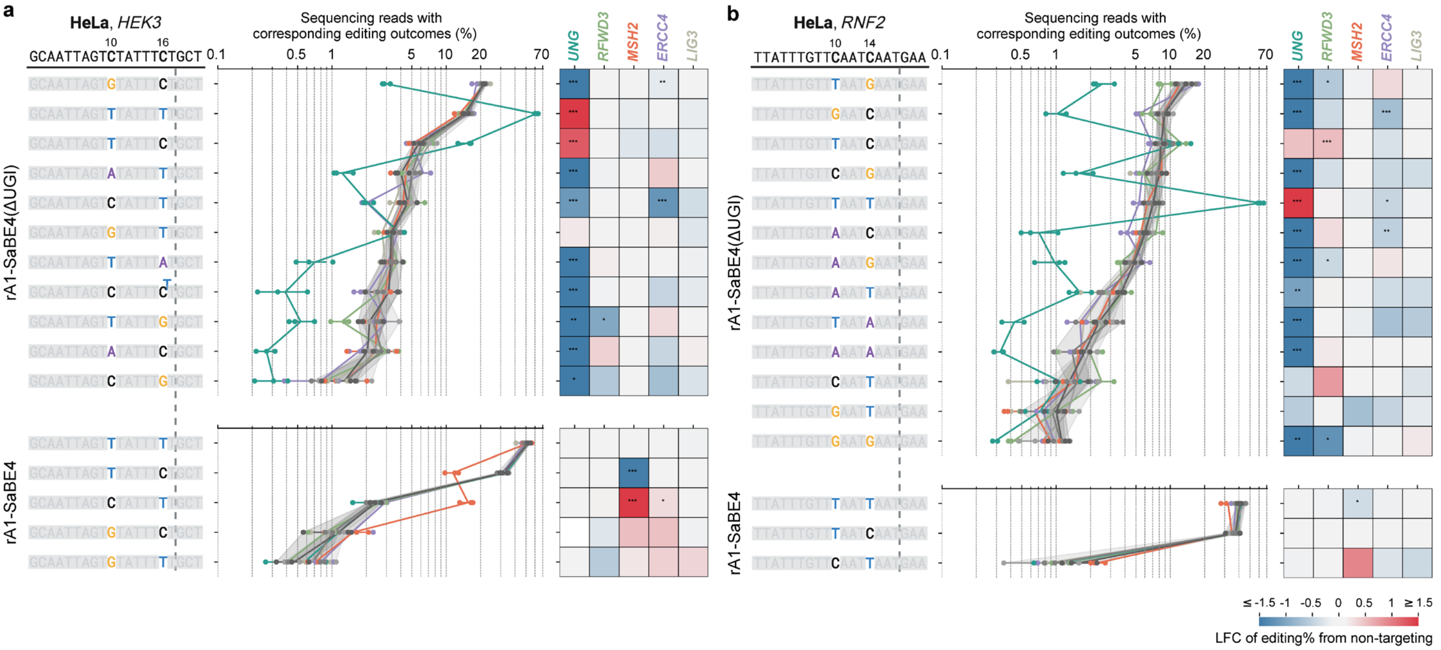

Supplementary Figure 15. Impacts of *UNG*, *RFWD3*, *MSH2*, *ERCC4*, and *LIG3* knockdown on all editing outcomes in HeLa cells. (**a-b**) Effects of indicated gene knockdown on editing outcomes at the *HEK3* (**a**) and *RNF2* (**b**) sites in HeLa cells. Protospacer sequences are shown at the top. Vertical dashed lines mark the Cas9-induced nick positions. Tract plots display absolute frequencies of editing outcomes upon specific gene knockdown (color-coded by gene as indicated). Three negative control samples without knockdown are shown in grey colors, with shaded areas representing ±2 STDs of mean. Dots show individual replicates, and tract lines show averages of n=3 biological replicates. Horizontal lines show ranges of the replicates for the corresponding outcomes. Heatmap shows $\log_2$ (fold change) (LFC) in frequencies of editing outcomes relative to the mean of negative controls. *: 1.5 < adjusted z-score < 2. **: 2< adjusted z-score < 3. ***: adjusted z-score > 3. Source data are provided as a Source Data file.

Supplementary Discussion:

Validation of CRISPRi screens

Following completion of the screens, the genomic DNA from the various cell populations was isolated, and ~5% of the genomic DNA was used as a template to amplify the *BFP* (C•G to T•A screen) or *dGFP* (C•G to G•C screen) locus. Editing efficiencies (C•G to T•A, C•G to G•C, and C•G to indels) were then quantified by NGS. Editing efficiencies of the target C in the unsorted control populations (bulk, +dox samples), as quantified with NGS, were similar to those as quantified by flow cytometry (Supplementary Figure 3). Furthermore, editing efficiencies of the target C in the sorted populations showed significant enrichment of the expected editing outcomes compared to the unsorted control populations. In addition, sequencing of the control arm (bulk, -dox samples) from the C•G to T•A screen revealed editing efficiencies lower than the limit of detection of NGS (0.1%), and that from the C•G to G•C screen showed minimal editing efficiencies (0.38% C•G to T•A and 0.13% C•G to G•C editing), indicating stringent doxycycline induction in our system. These data indicate efficient dox-induced cytosine base editing activity, and effective enrichment of our editing outcomes of interest following FACS.

The CRISPRi Sp-sgRNA region from each population of cells was amplified and sequenced via NGS at a sequencing depth of >200X per sgRNA (Supplementary Figure 4a-d). To validate the screens by monitoring dropout of essential genes, we compared the CRISPRi Sp-sgRNA distributions in the unsorted experimental arm (bulk, +dox samples) on day 13 with the initial CRISPRi Sp-sgRNA distributions on day 0 for each screen. For the C•G to T•A screen, we found that CRISPRi Sp-sgRNAs targeting essential genes from both replicates exhibited median $\log_2$ fold changes of -1.13 and -0.82, respectively, and were significantly more depleted than non-targeting CRISPRi Sp-sgRNAs and Sp-sgRNAs targeting non-essential genes (Supplementary Figure 4e). Sp-sgRNAs targeting essential genes in the C•G to G•C screen had median $\log_2$ fold changes of -1.82 and -2.02 for the two replicates, demonstrating a stronger and more robust depletion than in the C•G to T•A screen (Supplementary Figure 4f). In addition, we observed good correlation (Pearson's R>0.7) between the $\log_2$ fold change of the Sp-sgRNAs between the two replicates from both screens (Supplementary Figure 4g-h). Overall, these observations suggest that both screens demonstrated effective gene knockdown.

Identification of genes that are synthetic lethal to CBE expression and/or activity

To identify genes that are synthetic lethal to CBE expression and/or activity, we compared the CRISPRi Sp-sgRNA distributions in the experimental arm unsorted cells (bulk, +dox samples) to that of the control arm unsorted cells (bulk, -dox samples) from the C•G to G•C screen, as this screen demonstrated a better knockdown efficiency (as evaluated by the median and distribution of $\log_2$ fold changes of the CRISPRi Sp-sgRNAs targeting essential genes, Supplementary Figure 4e-f). To confirm dropout of essential genes, the CRISPRi Sp-sgRNA distributions in the control arm unsorted cells (bulk, -dox samples) were compared to that of the day 0 cells. We observed strong depletion of Sp-sgRNAs targeting essential genes (the median $\log_2$ fold changes of the CRISPRi Sp-sgRNAs targeting essential genes of the two replicates were -1.50 and -1.52, Supplementary Figure 16a) and good sample correlation (Pearson's r = 0.884, Supplementary Figure 16b). However, we observed a low correlation between the two replicates when the experimental arm unsorted cells (bulk, +dox samples) were compared with the control arm unsorted cells (bulk, -dox samples) (Pearson's r = 0.140, Supplementary Figure 16c). Because we consistently observed strong depletion of Sp-sgRNAs targeting essential genes in each replicate of both arms (Supplementary Figure 4f and Supplementary Figure 16a), a low correlation when comparing these two arms is not indicative of low screen quality.[4] Instead, we believe this is due to only a small number of genes that can single-handedly impact cell survival due to CBE expression and/or activity. Consequently, we observed a small number of genes whose sgRNAs were consistently depleted or enriched in both replicates.[5]

We then used the MAGeCK RRA algorithm to identify hits (Supplementary Figure 16d, full list in Supplementary Data 7).[6,7] A gene ontology biological process (GOBP) analysis of the data revealed that sgRNAs targeting genes involved in DNA replication (P = 8.74×10^{-7}) were significantly depleted in the population with CBE expressed

(bulk, +dox samples).[14] In particular, sgRNAs targeting genes involved in the G1/S phase transition (*HINFP*, *GMNN*, *PRIM2*, *MCM10*, *RPA2*, *MCM3*, *POLE*, and *MCM5*) and nucleosome assembly (*CHAF1A*, *CHAF1B*, and *RBBP4*) were depleted. Among the most statistically significantly depleted sgRNAs were those targeting *SUPT16H*, which encodes for the SPT16 protein, a subunit of the Facilitates Chromatin Transcription (FACT) complex. The FACT complex has been shown to displace Cas9 from genomic DNA, and thus is likely involved in the displacement of CBE from genomic DNA as well.[8] This result suggests that displacement of CBE from genomic DNA may be important for maintaining cell viability. An additional depleted gene was *HUWE1*, which encodes for the HUWE1 enzyme, an E3 ubiquitin-protein ligase that mediates ubiquitination of Polymerase Beta (POLB), a DNA polymerase involved in base excision repair.[9] HUWE1 also directly ubiquitinates p53 and cell division cycle 6 (cdc6), through which it regulates cell cycle arrest after DNA damage.[10,11] HUWEI's role in initiating cell cycle arrest after DNA damage may therefore be necessary for the cell to process the CBE intermediate rather than undergo apoptosis. Interestingly, all components of the MBD1-ATF7IP/2-SETDB1 complex, which is responsible for H3K9 trimethylation (and thus gene repression), were strongly depleted.[12] This complex may be involved in silencing the expression of CBE, and therefore may suggest that CBE expression is somewhat toxic to the cell. Determining the exact roles and therapeutic implications of these genes with respect to CBE expression and activity requires further investigation and is beyond the scope of this work.

Further discussion on certain hits below the z-score threshold

While there is evidence indicating that base editing can trigger DNA damage responses,[13] knock down of most of the DNA damage response (DDR) and cell cycle checkpoint genes (*ATR*, *ATM*, *HUS1, HUS1B, RAD1* and *CDK1*) that were hits in our screen did not significantly impact C•G to T•A or C•G to G•C editing efficiencies (Supplementary Figures 7-8). However, knockdown of *TIMELESS* (essential for S phase progression) and *RAD51* (essential for G2/M phase progression), which were found to be enriched in the GFP+ population in the C•G to T•A screen, decreased the C•G to T•A outcome by 1.7± 0.2 and 1.4 ± 0.1-fold, respectively (Supplementary Figure 7).[14,15] Their knockdown also led to notable cell growth defects. The knockdown of *APEX1*, *POLB* and *XRCC1*, BER genes that were not screen hits but we chose for validation as previously described, did not impact editing outcomes either. This suggests that downstream BER processing after uracil excision is not one of the primary pathways involved in resolving base editing intermediates, or there are redundant genes that can fulfill these roles. Knockdown of the two error-prone polymerases (*REV1* and *POLH*) only marginally decreased the C•G to G•C outcome, in agreement with previous studies.[16] While the REV1 polymerase plays a critical role in inducing C•G to G•C mutations during somatic hypermutation, the C•G to G•C outcome in cytosine base editing seems to occur through a REV1-independent pathway.[17,18]

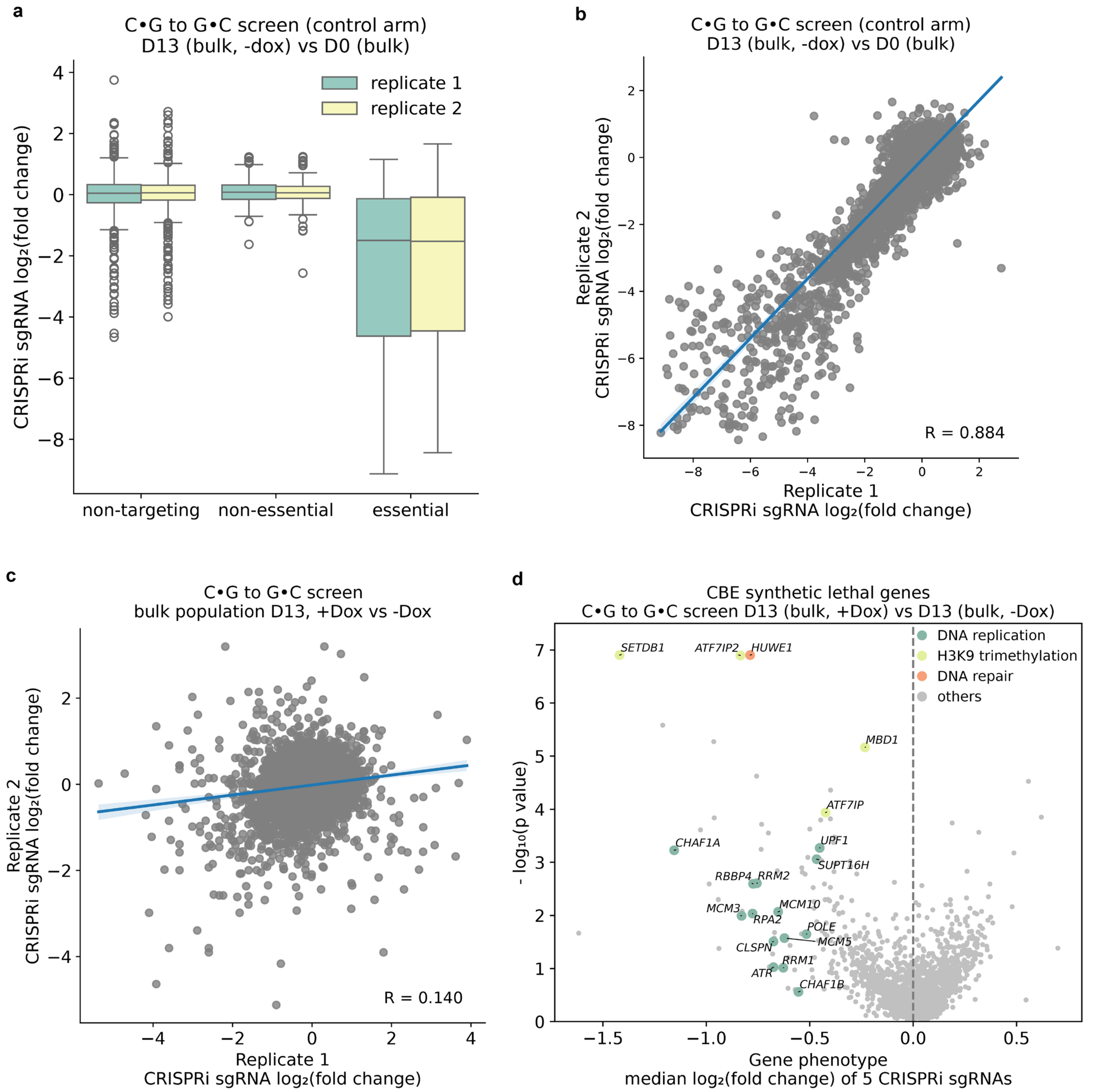

Supplementary Figure 16. Identification of genes that are synthetic lethal to CBE expression and/or activity. Screens were run as described in Figure 1c. **(a)** Shown in each boxplot are the distributions of $\log_2$ fold changes for CRISPRi sgRNAs classified by essentiality in each replicate of the C•G to G•C screen control arm, comparing bulk samples without dox induction (-Dox) on day 13 (D13) to their corresponding initial day 0 (D0) samples. **(b)** Shown in the scatterplot is the correlation between the $\log_2$ fold changes of CRISPRi sgRNAs of the two replicates in the C•G to G•C screen control arm, comparing bulk samples without dox induction (-Dox) on day 13 (D13) to their corresponding initial day 0 (D0) samples. **(c)** Shown in the scatterplot is the correlation between the $\log_2$ fold changes of CRISPRi sgRNAs of the two replicates, comparing the bulk samples from the C•G to G•C screen experimental arm (+Dox) on D13 to the control arm (-Dox) on D13. **(d)** The genes which were enriched or depleted upon induction of CBE expression from n = 2 independent replicates are shown in the volcano plot. Representative genes of the enriched pathways identified from GOBP analysis are highlighted in

colors indicated in the figure legends. Gene phenotypes (median log2 fold change of 5 CRISPRi sgRNAs) and *P* values were calculated using the MAGeCK RRA. Source data are provided as a Source Data file.

Supplementary References:

1. Horlbeck, M. A. *et al.* Compact and highly active next-generation libraries for CRISPR-mediated gene repression and activation. *eLife* **5**, e19760 (2016).
2. Richardson, C. D. *et al.* CRISPR–Cas9 genome editing in human cells occurs via the Fanconi anemia pathway. *Nat Genet* **50**, 1132–1139 (2018).
3. Hart, T., Brown, K. R., Sircoulomb, F., Rottapel, R. & Moffat, J. Measuring error rates in genomic perturbation screens: gold standards for human functional genomics. *Mol Syst Biol* **10**, 733 (2014).
4. Hanna, R. E. & Doench, J. G. Design and analysis of CRISPR–Cas experiments. *Nat Biotechnol* **38**, 813–823 (2020).
5. Doench, J. G. Am I ready for CRISPR? A user's guide to genetic screens. *Nat Rev Genet* **19**, 67–80 (2018).
6. Li, W. *et al.* MAGeCK enables robust identification of essential genes from genome-scale CRISPR/Cas9 knockout screens. *Genome Biol* **15**, 554 (2014).
7. Wang, B. *et al.* Integrative analysis of pooled CRISPR genetic screens using MAGeCKFlute. *Nat Protoc* **14**, 756–780 (2019).
8. Wang, A. S. *et al.* The Histone Chaperone FACT Induces Cas9 Multi-turnover Behavior and Modifies Genome Manipulation in Human Cells. *Molecular Cell* **79**, 221-233.e5 (2020).
9. Parsons, J. L. *et al.* Ubiquitin ligase ARF-BP1/Mule modulates base excision repair. *EMBO J* **28**, 3207–3215 (2009).
10. Chen, D. *et al.* ARF-BP1/Mule Is a Critical Mediator of the ARF Tumor Suppressor. *Cell* **121**, 1071–1083 (2005).
11. Hall, J. R. *et al.* Cdc6 Stability Is Regulated by the Huwe1 Ubiquitin Ligase after DNA Damage. *MBoC* **18**, 3340–3350 (2007).
12. Ichimura, T. *et al.* Transcriptional Repression and Heterochromatin Formation by MBD1 and MCAF/AM Family Proteins. *Journal of Biological Chemistry* **280**, 13928–13935 (2005).
13. Wang, X. *et al.* Cas12a Base Editors Induce Efficient and Specific Editing with Low DNA Damage Response. *Cell Reports* **31**, 107723 (2020).
14. Patel, J. A. & Kim, H. The TIMELESS effort for timely DNA replication and protection. *Cell. Mol. Life Sci.* **80**, 84 (2023).

15. Yoon, S.-W., Kim, D.-K., Kim, K. P. & Park, K.-S. Rad51 Regulates Cell Cycle Progression by Preserving G2/M Transition in Mouse Embryonic Stem Cells. *Stem Cells and Development* **23**, 2700–2711 (2014).

16. Arbab, M. *et al.* Determinants of Base Editing Outcomes from Target Library Analysis and Machine Learning. *Cell* **182**, 463-480.e30 (2020).

17. Masuda, K. *et al.* A Critical Role for REV1 in Regulating the Induction of C:G Transitions and A:T Mutations during Ig Gene Hypermutation. *J Immunol* **183**, 1846–1850 (2009).

18. Ulrich, H. D. Regulating post-translational modifications of the eukaryotic replication clamp PCNA. *DNA Repair* **8**, 461–469 (2009).
