# Supplementary material for: Elucidating the genetic mechanisms governing cytosine base editing outcomes through CRISPRi screens
Source: Nat Commun. 2025 May 20;16:4685. doi: 10.1038/s41467-025-59948-z (PMC12092667; doi:10.1038/s41467-025-59948-z)
Supplement: Supplementary file 2 — Description of Additional Supplementary Files [file 41467_2025_59948_MOESM2_ESM.pdf]

Description of Additional Supplementary Files for:

Elucidating the genetic mechanisms governing cytosine base editing outcomes through CRISPRi screens

Sifeng Gu, Zsolt Bodai, Rachel A. Anderson, Hei Yu Annika So, Quinn T. Cowan, and Alexis C. Komor

File Name: Supplementary Data 1
Description: Sequences of the sgRNA library used in screens

File Name: Supplementary Data 2
Description: Processed data (via MAGeCK RRA) from comparison of the CRISPRi Sp-sgRNAs in the GFP+ population to that of the pre-sorted bulk population (bulk, +dox sample) in the C•G to T•A screen to identify genes influencing C•G to T•A outcome.

File Name: Supplementary Data 3
Description: Processed data (via MAGeCK RRA) from comparison of the CRISPRi Sp-sgRNAs in the GFP+ population to that of the pre-sorted bulk population (bulk, +dox sample) in the C•G to G•C screen to identify genes influencing C•G to G•C outcome.

File Name: Supplementary Data 4
Description: List of names and descriptions of plasmids used in this work.

File Name: Supplementary Data 5
Description: List of plasmid names and sgRNA sequences of lentiviral constructs used for validation of screen hits.

File Name: Supplementary Data 6
Description: Primary sequences of key oligonucleotides and amino acids used in this work.

File Name: Supplementary Data 7
Description: Processed data (via MAGeCK RRA) from comparison of the CRISPRi Sp-sgRNA distributions in the experimental arm unsorted cells (bulk, +dox samples) to that of the control arm unsorted cells (bulk, -dox samples) from the C•G to G•C screen to identify genes that are synthetic lethal to CBE expression and/or activity.
