# Supplementary material for: Elucidating the genetic mechanisms governing cytosine base editing outcomes through CRISPRi screens
Source: Nat Commun. 2025 May 20;16:4685. doi: 10.1038/s41467-025-59948-z (PMC12092667; doi:10.1038/s41467-025-59948-z)
Supplement: Supplementary file 4 — Reporting Summary [file 41467_2025_59948_MOESM4_ESM.pdf]

nature portfolio

Corresponding author(s): Komor, Alexis C.

Last updated by author(s): Apr 30, 2025

Reporting Summary

Nature Portfolio wishes to improve the reproducibility of the work that we publish. This form provides structure for consistency and transparency in reporting. For further information on Nature Portfolio policies, see our Editorial Policies and the Editorial Policy Checklist.

Statistics

For all statistical analyses, confirm that the following items are present in the figure legend, table legend, main text, or Methods section.

n/a	Confirmed	
☐	☒	The exact sample size (*n*) for each experimental group/condition, given as a discrete number and unit of measurement
☐	☒	A statement on whether measurements were taken from distinct samples or whether the same sample was measured repeatedly
☐	☒	The statistical test(s) used AND whether they are one- or two-sided<br>*Only common tests should be described solely by name; describe more complex techniques in the Methods section.*
☒	☐	A description of all covariates tested
☒	☐	A description of any assumptions or corrections, such as tests of normality and adjustment for multiple comparisons
☐	☒	A full description of the statistical parameters including central tendency (e.g. means) or other basic estimates (e.g. regression coefficient) AND variation (e.g. standard deviation) or associated estimates of uncertainty (e.g. confidence intervals)
☐	☒	For null hypothesis testing, the test statistic (e.g. *F*, *t*, *r*) with confidence intervals, effect sizes, degrees of freedom and *P* value noted<br>*Give P values as exact values whenever suitable.*
☒	☐	For Bayesian analysis, information on the choice of priors and Markov chain Monte Carlo settings
☒	☐	For hierarchical and complex designs, identification of the appropriate level for tests and full reporting of outcomes
☐	☒	Estimates of effect sizes (e.g. Cohen's *d*, Pearson's *r*), indicating how they were calculated

Our web collection on statistics for biologists contains articles on many of the points above.

Software and code

Policy information about availability of computer code

Data collection	Illumina MiniSeq control software was used to collect next-generation sequencing data.
Data analysis	CRISPResso2 (version 2.0.20b)–https://github.com/pinellolab/CRISPResso2<br>Python 3.9.5<br>MAGeCK v0.5.9.1<br>Pandas v1.4.2<br>Matplotlib v3.4.3<br>Seaborn v0.11.2<br>Biorad ProSort software v1.6 (S3e Cell Sorter)<br>Flowjo v10.9

For manuscripts utilizing custom algorithms or software that are central to the research but not yet described in published literature, software must be made available to editors and reviewers. We strongly encourage code deposition in a community repository (e.g. GitHub). See the Nature Portfolio guidelines for submitting code & software for further information.

Data

Policy information about availability of data

All manuscripts must include a data availability statement. This statement should provide the following information, where applicable:

- Accession codes, unique identifiers, or web links for publicly available datasets
- A description of any restrictions on data availability
- For clinical datasets or third party data, please ensure that the statement adheres to our policy

Next-generation sequencing data are available on the NCBI Sequencing Read Archive database under project number PRJNA1081436. Plasmids from this study will be available at Addgene.

Research involving human participants, their data, or biological material

Policy information about studies with human participants or human data. See also policy information about sex, gender (identity/presentation), and sexual orientation and race, ethnicity and racism.

Reporting on sex and gender	NA
Reporting on race, ethnicity, or other socially relevant groupings	NA
Population characteristics	NA
Recruitment	NA
Ethics oversight	NA

Note that full information on the approval of the study protocol must also be provided in the manuscript.

Field-specific reporting

Please select the one below that is the best fit for your research. If you are not sure, read the appropriate sections before making your selection.

☒ Life sciences ☐ Behavioural & social sciences ☐ Ecological, evolutionary & environmental sciences

For a reference copy of the document with all sections, see nature.com/documents/nr-reporting-summary-flat.pdf

Life sciences study design

All studies must disclose on these points even when the disclosure is negative.

Sample size	No statistical methods were used to predetermine sample size. Sample sizes were determined (n = 2 to 5) based on literature precedence for genome editing experiments.
Data exclusions	No data was excluded from this study.
Replication	All experiments were performed as two or more independent biological replicates, as specified in each figure caption. All replications were successful.
Randomization	No randomization was used; human cells were treated identically throughout experiments.
Blinding	No blinding was used; human cells were treated identically throughout experiments.

Reporting for specific materials, systems and methods

We require information from authors about some types of materials, experimental systems and methods used in many studies. Here, indicate whether each material, system or method listed is relevant to your study. If you are not sure if a list item applies to your research, read the appropriate section before selecting a response.

Materials & experimental systems

n/a	Involved in the study	
☒	☐	Antibodies
☐	☒	Eukaryotic cell lines
☒	☐	Palaeontology and archaeology
☒	☐	Animals and other organisms
☒	☐	Clinical data
☒	☐	Dual use research of concern
☒	☐	Plants

Methods

n/a	Involved in the study	
☒	☐	ChIP-seq
☐	☒	Flow cytometry
☒	☐	MRI-based neuroimaging

Eukaryotic cell lines

Policy information about cell lines and Sex and Gender in Research

Cell line source(s)	HEK293T (CRL-3216), HeLa (CCL-2), K562(CCL-243) cells from American Type Cell Culture Collection (ATCC)
Authentication	Authenticated by supplier with short tandem repeat (STR) profiling
Mycoplasma contamination	Cells were tested both by ATCC and our lab for mycoplasma contamination, and all tests were negative for contamination.
Commonly misidentified lines (See ICLAC register)	No commonly misidentified cell lines were used in this study.

Plants

Seed stocks	NA
Novel plant genotypes	NA
Authentication	NA

Flow Cytometry

Plots

Confirm that:

☒ The axis labels state the marker and fluorochrome used (e.g. CD4-FITC).

☒ The axis scales are clearly visible. Include numbers along axes only for bottom left plot of group (a 'group' is an analysis of identical markers).

☒ All plots are contour plots with outliers or pseudocolor plots.

☒ A numerical value for number of cells or percentage (with statistics) is provided.

Methodology

Sample preparation	For K562 cells, cells were precipitated and resuspended in PBS at around 1.5M/ml. For HEK293T and HeLa cells, cells were washed with PBS, detached by adding Accumax, and resuspended in sort buffer (PBS, 2.5 mM EDTA, 25 mM HEPES, 1% FBS, 1% PS).
Instrument	Biorad S3e cell sorter, BD FACSAria II, BD FACSAria Fusion, BD LSRFortessa X-20
Software	Flowjo v10.9
Cell population abundance	For analytical flow cytometry, > 10000 cells were collected for each sample. For arrayed validation experiments, > 5000 cells were collected for sequencing. For pooled screen, 30M cells were sorted into different populations.

Gating strategy

The instruments were calibrated and quality control checked before each FACS experiment. No color (untransfected cells) and single color (e.g. GFP or mCherry transfected cells) control populations were used to set up gating. Doublets were gated out using forward and side scattering width against area. See Supplementary Figure 3 for an example.

☒ Tick this box to confirm that a figure exemplifying the gating strategy is provided in the Supplementary Information.
